# Protein Nucleotidylylation in +ssRNA Viruses

**DOI:** 10.3390/v13081549

**Published:** 2021-08-05

**Authors:** Alice-Roza Eruera, Alice M. McSweeney, Geena M. McKenzie-Goldsmith, Vernon K. Ward

**Affiliations:** Department of Microbiology & Immunology, School of Biomedical Sciences, University of Otago, PO Box 56, Dunedin 9054, New Zealand; alice.eruera@postgrad.otago.ac.nz (A.-R.E.); alice.mcsweeney@otago.ac.nz (A.M.M.); geena.mckenzie-goldsmith@postgrad.otago.ac.nz (G.M.M.-G.)

**Keywords:** nucleotidylylation, VPg, RdRp, calicivirus, picornavirus, potyvirus, coronavirus

## Abstract

Nucleotidylylation is a post-transcriptional modification important for replication in the picornavirus supergroup of RNA viruses, including members of the *Caliciviridae*, *Coronaviridae*, *Picornaviridae* and *Potyviridae* virus families. This modification occurs when the RNA-dependent RNA polymerase (RdRp) attaches one or more nucleotides to a target protein through a nucleotidyl-transferase reaction. The most characterized nucleotidylylation target is VPg (viral protein genome-linked), a protein linked to the 5′ end of the genome in *Caliciviridae*, *Picornaviridae* and *Potyviridae*. The nucleotidylylation of VPg by RdRp is a critical step for the VPg protein to act as a primer for genome replication and, in *Caliciviridae* and *Potyviridae,* for the initiation of translation. In contrast, *Coronaviridae* do not express a VPg protein, but the nucleotidylylation of proteins involved in replication initiation is critical for genome replication. Furthermore, the RdRp proteins of the viruses that perform nucleotidylylation are themselves nucleotidylylated, and in the case of coronavirus, this has been shown to be essential for viral replication. This review focuses on nucleotidylylation within the picornavirus supergroup of viruses, including the proteins that are modified, what is known about the nucleotidylylation process and the roles that these modifications have in the viral life cycle.

## 1. Introduction

Positive-sense single-stranded (+ss) RNA viruses are an enormously diverse class of animal, plant and bacterial viruses. Despite this diversity, +ssRNA viruses share a number of key features which facilitate their successful propagation. Notably, the ubiquitous RNA-dependent RNA polymerase is conserved across all +ssRNA viruses, as are its associated functions, targets, main structural architecture and seven highly conserved functional motifs [1,2,3]. RNA polymerases are multifunctional proteins primarily involved in RNA synthesis and transcription of the viral genome (and in some cases the sub-genome), as directed by an RNA template. In addition to transcription, RNA polymerases play key roles in modulating the activity of other molecules, including post-translational modification by nucleotidylylation.

The attachment of one or more oligonucleotides, such as a GTP (guanylylation) or UTP (uridylylation) to a target protein, can influence the function of the recipient enzyme or protein. Nucleotidylylation of viral proteins by RNA polymerases is therefore a mechanism by which viruses can regulate protein functions during the viral lifecycle. In addition, RNA polymerases implicated in the nucleotidylylation of other viral proteins have also been shown to be nucleotidylylated.

Analysis of positive-strand genomic RNA synthesis by a range of +ssRNA viruses shows that initiation can occur via a protein primer-dependent mechanism regulated by the nucleotidylylation of a VPg protein (viral protein genome-linked) [4,5,6,7]. While picornavirus VPg is only involved in genome replication, the nucleotidylylation of calicivirus and potyvirus VPg and its consequent association with the 5′ end of +ssRNA viral genomes—and in some cases, subgenomic RNA—is an essential step for viral protein synthesis, playing a critical role in translation initiation through interaction with cellular translation initiation factors [8,9,10,11,12].

The biochemical modification of VPg that precedes VPg-mediated priming of viral genome replication, and to a lesser extent the nucleotidylylation of the polymerase itself, has been the basis of a body of research across a number of +ssRNA viral families and is the focus of this review. The picornaviruses represent the most widely studied viral family for nucleotidylylation of VPg proteins, with extensive information for both the biochemical nature of the reaction and the accessory components required for efficient nucleotidylylation. In contrast, less is known about VPg nucleotidylylation in the *Caliciviridae* and *Potyviridae* families, and the details of nucleotidylylation in astroviruses and sobemoviruses remain to be elucidated. Viruses within the order *Nidovirales* also undertake protein nucleotidylylation; however, they do not encode a VPg protein, nor has a direct role for the nucleotidylylated proteins in priming genome replication been described.

**Figure 1:**
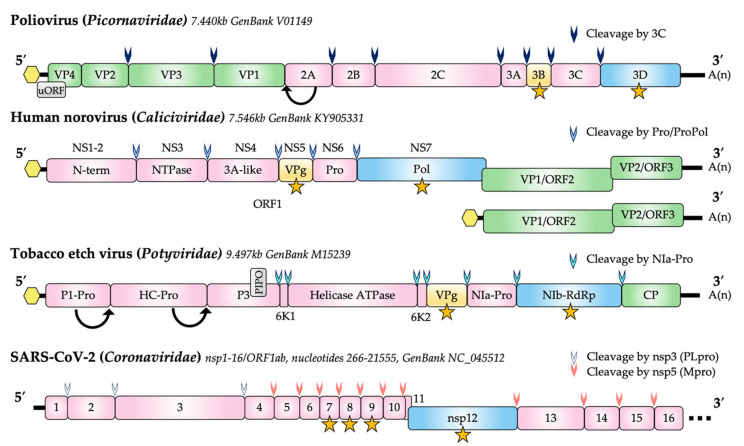
Genome structure of representative members of the *Caliciviridae, Potyviridae, Picornaviridae* and *Coronaviridae*. The presence of a covalently attached (nucleotidylylated) 5′ VPg is shown as a yellow hexagon. The coding region for VPg proteins is shown in yellow and proteins known to be nucleotidylylated are indicated by a yellow star. Proteolytic processing of viral polyproteins are indicated by arrowheads. Potyvirus P1-Pro and HC-Pro self-cleave from the polyprotein (arrows). Viral capsid proteins are shown in green and the RNA-dependent RNA polymerases (RdRp) are shown in blue. Non-structural proteins (nsp) other than RdRp and VPg are shown in pink with the common name and/or nsp designation indicated. A second reading frame (uORF) produced by poliovirus is shown in grey. The PIPO coding region expressed as a fusion to P3 via a polymerase slippage mechanism is shown in grey for tobacco etch virus. In caliciviruses, the protease (pro) and polymerase (RdRp) are found as unprocessed precursors, analogous to 3CD in picornaviruses. The NS and VP1 genes of some calicivirus genera (e.g., lagoviruses) are a single ORF. Nucleotide lengths for the genomes depicted are indicated and genomes are not to scale.

Regardless of the exact mechanism, nucleotidylylation is essential to the replication of a wide range of +ssRNA viruses, and this review will focus upon our understanding of the function and importance of nucleotidylylation during the life cycle of RNA viruses belonging primarily to the *Caliciviridae*, *Coronaviridae*, *Picornaviridae* and *Potyviridae* viral families (Figure 1).

## 2. RNA Polymerases and Nucleotidylylation

Viral RNA polymerases primarily function to catalyze the transcription of RNA during viral replication. The RNA polymerases of picornaviruses, caliciviruses, potyviruses, sobemoviruses and coronaviruses are supergroup I RdRps [13] and all cluster within branch 2 of the ‘picornavirus supergroup’ based on RdRp phylogeny as defined by Wolf et al. [14]. The common and unique features of RNA polymerases are reviewed in Velthuis [2] and RdRp structures are reviewed by Ferrero et al. [15]. We refer the reader to these reviews for complementary information.

Nucleotidyl-transferases are a diverse group of enzymes that transfer nucleotides to a protein, nucleic acid or other molecule. Enzymes with nucleotidyl-transferase activity are involved in antibiotic resistance [16], polyadenylation of RNA [17], DNA repair [18], regulation of protein activity [19,20] and viral replication [21].

Nucleotidyl-transferase or nucleotidylylation reactions, catalyzed by the viral polymerase, have been shown to be essential for the replication of certain classes of virus. These viruses use a protein, VPg, covalently linked to a nucleotide monophosphate (NMP) to prime replication of the viral genome. A tyrosine residue in VPg provides the free hydroxyl for the nucleotidylylation reaction in the *Astroviridae*, *Caliciviridae*, *Picornaviridae* and *Potyviridae* families, while the *Sobemovirus* genus uses a threonine or a serine amino acid for nucleotidylylation [22,23,24,25,26,27,28]. The role of VPg in priming RNA synthesis has not been confirmed for sobemoviruses [29].

**Figure 2:**
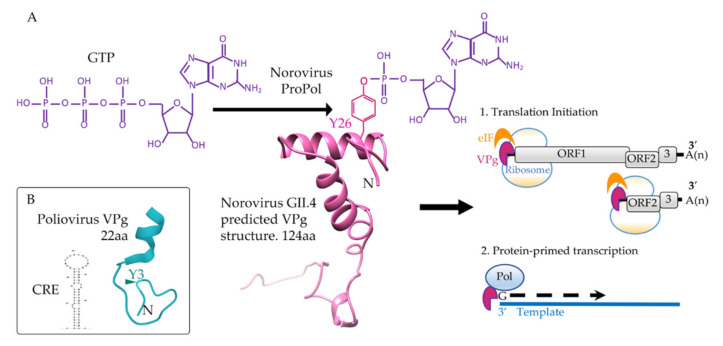
Guanylylation of a calicivirus VPg. (**A**) For the nucleotidylylation reaction, ProPol catalyzes a nucleophilic attack by the hydroxyl of Y26 of VPg (pink) on the alpha phosphate of GTP (purple). This produces a phosphodiester bond covalently linking GMP onto Y26 of VPg. Guanylylated human norovirus VPg functions to recruit host eukaryotic initiation factors (eIF) for translation and interacts with Pol or ProPol to stimulate protein-primed transcription of the RNA genome. Structural predictions for human norovirus GII.4 VPg (KY905331) were performed with Phyre and imaged with Chimera [33,34]. (**B**) In addition to polymerase, a CRE RNA (AY184219) is required for nucleotidylylation of the poliovirus VPg (blue) on tyrosine 3 (Y3). The poliovirus 1 VPg structure (2BBL) was obtained from PDB and the representation of the CRE RNA stem-loop predicted using mFold [35]. Nucleotidylylation of poliovirus VPg does not contribute to translation initiation, only protein-primed transcription. Amino acid (aa) length of the VPg proteins is indicated.

Catalysis of NTPs to NMPs by polymerases occurs via a two-metal-ion mechanism that is reliant on the presence of divalent cations in the form of Mg^2+^ or Mn^2+^ [30,31,32]. In the first step of the reaction, a nucleotide, VPg and two divalent cations bind to the polymerase. The polymerase then undergoes a conformational change to orientate and retain the NTP for catalysis. In the nucleotidylylation reaction, one metal ion functions to lower the pK_a_ of the hydroxyl group (in this instance, on the tyrosine or threonine of VPg), making it an effective nucleophile. The hydroxyl group then performs a nucleophilic attack on the alpha phosphate of an NTP. The result is the formation of a phosphodiester bond between the nucleophile and the alpha phosphate and protonation of a pyrophosphate molecule, a by-product of the reaction (Figure 2).

Nucleotidylylation reactions utilizing asparagine, lysine and possibly serine have also been described for viruses in the *Coronaviridae* family [36,37]. Lysine and asparagine contain a free amino group rather than a hydroxyl group, and therefore, the resulting bond is a phosphoramide rather than a phosphodiester. In the *Coronaviridae* family, nucleotidylylation occurs on selected proteins in the replication complex (nsp 7, 8, 9 and 12 for SARS-CoV-2), although the exact biological role is not known [36,37].

## 3. Picornaviruses

The *Picornaviridae* family comprises a large group of non-enveloped positive-sense single-stranded RNA viruses. The genome is translated using an internal ribosomal entry site (IRES) and produces a large polyprotein that is cleaved to its constituent proteins (Figure 1). For enteroviruses, a second ORF (termed uORF) has been identified [38]. Currently, the family contains 63 genera which is subsequently divided into 147 species [39]. Viruses in this family are primarily transmitted by fecal–oral or respiratory routes and infect mammals, birds, reptiles, amphibians and fish [39]. Of these viruses, the *Enterovirus* genus contains a number of important human pathogens including coxsackieviruses, polioviruses, echoviruses, enteroviruses and rhinoviruses. Although severe disease (such as meningitis and paralysis) does occur, a vast majority of infections are asymptomatic or only cause mild infection [40]. The abundance of these viruses in the environment means that they present a significant annual burden to society in the forms of morbidity, mortality, health care costs and economic losses [40,41,42]. Aside from humans, picornaviruses also cause significant infections in livestock with substantial economic burdens. For example, foot and mouth disease virus (FMDV) has a significant impact in Asian and African countries and is highly contagious among cloven-hoofed animals [43].

### 3.1. Picornavirus Nucleotidylylation and Replication

The replication of picornaviruses has been reviewed recently [40], and for a comprehensive review of protein-primed replication, the reader is referred to [44]. As with caliciviruses and potyviruses, the VPg (3B) protein is a central player in replication of picornaviruses; it was first described for poliovirus in 1977 upon discovery of a protein covalently attached to the 5′ end of the viral genome [45]. VPg proteins have subsequently been detected in all viruses in the family and are a key determinant for classing new viruses into *Picornaviridae* [39]. Both positive-sense and negative-sense RNA can be isolated with VPg (3B) covalently attached [46,47], and the nucleotidylylation of VPg by the viral polymerase (3D) is an essential step leading to the incorporation of VPg onto the viral genome.

Picornaviruses have been shown to hijack a host protein, TDP2, to cleave the phosphodiester bond linking VPg to the genomic RNA, with this RNA becoming the template for translation [48,49,50]. In addition to a polymerase, viruses in the picornavirus family require cis-acting RNA elements for VPg nucleotidylylation and replication of the viral genome. These include the 5′ clover leaf (5′CL) [51,52,53,54]; the 3′ untranslated region (3′ UTR), including the poly (A) tail [55,56]; and a cis replication element (CRE) in the open reading frame [46,57].

Viruses in the *Picornaviridae* encode one VPg protein, with the exception of FMDV, which encodes three, all of which can be uridylylated [58,59]. VPg proteins in this family are small (~19–26 amino acids in length) with a conserved tyrosine at position three of the amino acid sequence [60] (Figure 2). This tyrosine is used for the covalent linkage of VPg to the RNA, through formation of the VPgpUpU primer. Structural analysis of poliovirus 1 VPg by NMR shows a flexible protein which, in the presence of trimethylamine N-oxide, forms a single conformer with an N-terminal loop containing the tyrosine for uridylylation and a C-terminal helix [61].

#### 3.1.1. Precursor Proteins Associated with Nucleotidylylation

The nucleotidylylation of VPg in picornaviruses involves a complex interplay of a range of viral components. Partially processed precursor proteins from the viral polyprotein play important roles in nucleotidylylation, including different forms of the VPg (3B) and the 3CD protein (protease and polymerase precursor protein).

Precursors of VPg include the 3AB and 3BC proteins, both of which have been proposed as sources of VPg for uridylylation [62,63,64]. In addition to being a target for uridylylation, 3AB stimulates the polymerase activity of 3D [65,66,67] and also functions as an RNA binding protein with RNA chaperone and helix destabilizing activities [66,67,68]. The RNA binding activity of 3AB alone is non-specific, but when complexed with 3CD, the binding becomes specific for the 5′CL and 3′UTR of picornaviruses [66,69]. Interaction with the 5′CL and 3′UTR helps to promote circularization of the genome and is important for replication of the positive-sense and negative-sense RNA [54,70,71,72,73,74].

Poliovirus 3CD lacks polymerase activity but retains protease activity, and interestingly, this protein can stimulate uridylylation by 100-fold in in vitro assays [58,75,76]. The ability of 3CD to bind either the 5′CL or CRE RNA is required for this stimulation and it has been hypothesized that 3CD might provide specificity to the uridylylation reaction by enhancing the binding of 3D and/or VPg to these RNA structures [66,67,75,77,78].

#### 3.1.2. Uridylylation of VPg

In picornaviruses, formation of VPgpUpU is reliant on the CRE, which functions as a template for the uridylylation reaction [57,79,80]. CREs have been identified within the open reading frames of cardioviruses [81], polioviruses [82], hepatovirus [83], parechovirus [84], and sapelovirus [85]. The CRE of FMDV was identified in the 5′UTR [86], whereas all others are within the first open reading frame. The 3D polymerase, VPg and uridine triphosphate (UTP) interact with the CRE, thus bringing all three components into close association for uridylylation [75,78,80,87]. 3D polymerase then catalyzes a reaction between the free hydroxyl of tyrosine three of VPg and UTP to form a phosphodiester bond [88].

Currently, a “slide-back” mechanism for uridylylation of VPg on the CRE RNA is proposed, which utilizes a conserved 5′AAACA 3′ motif, representing nucleotides five through nine of the CRE RNA loop [79,87]. For poliovirus, this model proposes that the linkage of UMP to tyrosine three of VPg is templated by adenine five (A5, the first nucleotide of the conserved motif) of the CRE loop. VPgpU then “slides back” and the covalently linked uracil hydrogen bonds with adenine six (the second nucleotide in the conserved motif), followed by the addition of a second uracil, again using A5 as the template [87]. Following the formation of VPgpUpU, elongation of the VPg primer by Pol ceases [87,89]. The precise mechanism for how the polymerase knows to abort has not been determined, but it has been hypothesized that it is due to a structural characteristic of Pol during the reaction.

Two binding sites have been described for VPg on the polymerase, leading to two different models for the uridylylation reaction. In the first of these, VPg binds to the ‘front’ of the polymerase and presents the conserved tyrosine for nucleotidylylation into the active site of Pol [90,91,92]. This has been observed in the FMDV crystal structure showing VPg bound to 3Dpol with tyrosine three in the active site of Pol, and suggests a cis mechanism for FMDV uridylylation [92].

In contrast, uridylylation of enterovirus 71 has been proposed to occur in trans as VPg binds to the polymerase at the base of the palm domain, termed site-311 [93]. Substitutions to amino acids within this site reduce uridylylation activity by more than 90% but do not affect RNA elongation activity, indicating that this site is independent from the RNA polymerization activity [93]. A trans-uridylylation mechanism for coxsackie B virus has also been proposed [94] and similar results have also been seen for poliovirus through structure predictions and biochemical experiments [95].

#### 3.1.3. Uridylylation of Non-VPg Proteins

Aside from VPg, other viral proteins and protein precursors are able to be uridylylated. In the presence of a 15 nucleotide adenylate template, 3Dpol and 3CD become uridylylated [96]. This uridylylation can be further stimulated by the addition of the 3AB precursor and can also be detected in poliovirus-infected HeLa cells [96]. The reaction occurs intermolecularly with a short template but switches to an intramolecular mechanism in the presence of poly (A) RNA. As with VPg, the linkage is thought to be through a phosphodiester bond, but the site(s) of uridylylation have not been able to be identified [96]. Whether there is a functional role in uridylylation of 3Dpol remains to be determined; however, phosphate chirality experiments showed that the 3Dpol uridylylates are not obligate intermediates in the formation of uridylylated VPg [96]. However, it should be noted that all of the RNA polymerases implicated in nucleotidylylation (Figure 1) are themselves nucleotidylylated, implying a yet to be determined role for this modification.

Uridylylation of 3BC and 3BCD can also be detected in in vitro reactions [64]. Mutation of the 3BC cleavage site results in viruses that are still able to replicate and forms 3BC-linked RNA, showing that this can be used to prime RNA synthesis [64,97]. In this system, uridylylation of 3BC showed a 10-fold greater efficiency than VPg alone and has led to the suggestion that 3BC is the precursor protein that is uridylylated on the CRE and subsequently processed to VPgpUpU [64]. 3BC uridylylation has also been observed for FMDV [58]. However, the 3BC protein is expressed at low levels during infection and normally cannot be observed in infected cells. Uridylylation of the VPg precursor 3AB has been detected in the presence of a short adenylate template with Mn^2+^ [96], but in the presence of CRE RNA and Mg^2+^, no uridylylation is detectable [64]. Whether 3AB is uridylylated during an infection is not clear.

#### 3.1.4. Replication of the Negative-Sense and Positive-Sense RNA

The overall picture that is emerging for the replication of picornavirus genomes is outlined below. The VPgpUpU formed on the CRE of the positive-sense RNA is transferred through an unknown mechanism to the 3′ end of the poly (A) tail of the positive-sense RNA. The polymerase can then extend the RNA using uridylylated VPg as the primer to produce the negative-sense viral RNA with VPg covalently attached to the 3′ end [98]. However, it is worth noting that there is some disagreement in the literature as to whether a CRE is required for the uridylylation of VPg, specifically for negative-sense RNA synthesis [46,57,99,100]. The poly (A) tail has been proposed as an alternative RNA structure to stimulate uridylylation in this instance [46,89].

For synthesis of the positive-sense RNA, the double-stranded replicative form of the RNA must be partially unwound or destabilized to allow uridylylated VPg to transfer to the 3′ end of the negative-sense RNA. As the negative-sense RNA begins with AA bases, uridylylated VPg base pairs with these and the polymerase again elongates the RNA using the negative-sense as a template, thus yielding positive-sense genomic RNA with VPg linked at the 5′ end.

Overall, this paints a complex picture of nucleotidylylation in vivo, encompassing both viral and host proteins in the presence of RNA to achieve efficient replication. Dissecting the role of individual components in relation to RNA replication remains an ongoing objective in the understanding of this complex process.

## 4. Caliciviruses

*Caliciviridae* is a family of +ssRNA viruses, comprising 11 viral genera that infect mammals (*Lagovirus*, *Norovirus*, *Nebovirus*, *Recovirus*, *Sapovirus*, *Valovirus* and *Vesivirus* genera), birds (*Bavovirus* and *Nacovirus* genera) and fish (*Minovirus* and *Salovirus* genera) [101], causing diseases which encompass respiratory, hemorrhagic and gastrointestinal pathologies. Unclassified caliciviruses have also been identified in amphibians, reptiles and lampreys. Representative caliciviruses include human noroviruses (HuNV; *Norovirus*), rabbit hemorrhagic disease virus (RHDV; *Lagovirus*), and feline calicivirus (FCV; *Vesivirus*).

Notably, noroviruses became the most common cause of viral gastroenteritis following the global introduction of rotavirus vaccines [102,103,104,105]. Causing an estimated 685 million cases per annum, a report published by the Center for Disease Control and Prevention in 2016 revealed that human noroviruses have an estimated medical and socioeconomic burden of $60 billion USD each year [106]. Norovirus is highly infectious and is responsible for around ~800,000 hospitalizations globally each year and more than 50% of all viral gastroenteritis outbreaks in the world [107,108,109]. Despite this, there remain no approved antiviral therapeutics or vaccines available for the treatment or prevention of noroviral infections.

### 4.1. Calicivirus Nucleotidylylation

Calicivirus genomes carry a VPg covalently bound to the 5′ end, whilst the 3′ end has a poly (A) tail. The genome usually encodes three open reading frames (ORF1–3), the first of which encodes all the non-structural proteins, whilst ORF2 and ORF3 encode the major and minor capsid proteins. The ORF1 non-structural gene is translated to a polyprotein which is post-translationally cleaved into precursor and mature proteins by a 3C-like protease (NS6) (Figure 1).

Within caliciviruses, the length of VPg proteins can vary from 65 amino acids (e.g., bovine nebovirus VPg) to 138 amino acids (e.g., Norwalk virus VPg) [26]. The structure of feline calicivirus and murine norovirus (MNV) VPg proteins were solved by nuclear magnetic resonance spectroscopy, revealing that the core of the calicivirus VPg protein is the only ordered domain, and is flanked by disordered N- and C-termini [110]. Regions of intrinsic disorder allow viral proteins to interact with a range of potential binding partners. Unlike picornavirus VPg proteins, calicivirus VPg proteins are known to recruit host cell translation machinery, namely eIF4E and eIF4G [9,111]. Recruitment of these initiation factors to the viral nucleic acid by VPg initiates translation. In addition to this role, VPg can also bind RNA polymerases and their precursors, induce a G1/S cell cycle arrest and has been proposed to serve as a protective cap for the viral genome against detection by the host immune system [8,27,112,113,114,115,116,117].

Nucleotidylylation is an essential step in the viral lifecycle of caliciviruses, with nucleotidylylated VPg acting to prime transcription of the positive-sense genome by viral RdRp in vitro [5,113] and initiate translation [5,26,111,113,118]. Experimental evidence for MNV, for which a robust cell model exists [119], demonstrated that all infectious MNV virions have VPg caps on the 5′ end of the genome [26], confirming the essential requirement of nucleotidylylated VPg in viral replication.

Nucleotidylylation of VPg by RNA polymerases, specifically the addition of GTP or UTP [27], facilitates the role of VPg in priming RNA synthesis, leading to covalent attachment of VPg at the 5′ end of the viral genome and/or sub-genome [120]. A mass spectrometry-based approach identified that VPg from FCV and MNV are covalently linked to guanosine via a tyrosine at position 24 and 26, respectively [26], that lies within a highly conserved acidic amino acid motif (DEEYD/EE). Guanylylation of VPg provides the 5′ G nucleotide of the viral genome, and hence, mutation of the tyrosine in this motif is detrimental to viral replication and results in a non-infectious virus [26]. The calicivirus VPg N-terminus adjacent to the nucleotidylylated tyrosine is rich in lysine/arginine amino acids and has been shown to have both nucleotide binding capability and a role in nucleotidylylation [121]. Deletion of this region, specifically the first 3, 8 or 10 amino acids of the N-terminus of HuNV VPg, resulted in a progressive reduction of nucleotidylylation at Tyr27 [27].

#### 4.1.1. Proteins That Catalyze the Nucleotidyl-Transferase Reaction

The biomolecular mechanisms behind nucleotidylylation in caliciviruses are not well understood. In contrast to poliovirus nucleotidylylation, where 3Dpol is the active form of the polymerase responsible for catalyzing the reaction, it has been shown for the *Caliciviridae* family that this is more variable. In MNV and HuNV, nucleotidylylation is catalyzed by both forms of polymerase, the mature RdRp and the ORF1-derived precursor protein comprised of unprocessed protease-polymerase (ProPol, analogous to picornavirus 3CD) [27,121]. In contrast, for FCV, the protease and polymerase remain as a single protein, hence nucleotidylylation relies solely on the 3CD equivalent [122,123]. ProPol was also found to be the predominant form of polymerase in RHDV infected cells [124,125]. In HuNV, ProPol has been demonstrated to be 100-fold more efficient at nucleotidylylation than the mature polymerase, and a similar finding has been shown for RHDV VPg [121]. This suggests that ProPol may be the primary form of polymerase performing protein nucleotidylylation in caliciviruses.

The ability of calicivirus RdRps to nucleotidylylate target proteins from other viruses varies. HuNV RdRp specifically nucleotidylylates only HuNV VPg; in contrast, MNV RdRp is able to nucleotidylylate both HuNV VPg and MNV VPg [126]. Interestingly, the MNV polymerase was more efficient at nucleotidylylating HuNV VPg than the HuNV RdRp, and both HuNV and MNV RdRps nucleotidylylated themselves in vitro [126]. Whether this self-nucleotidylylation mechanism is similar to that identified in coronaviruses and picornaviruses is not known, nor have the target residue(s) been identified and nor has it been determined whether this is an essential function for viral replication.

#### 4.1.2. The Role of RNA in Nucleotidylylation

The evidence for RNA structures contributing to the nucleotidylylation of calicivirus VPg proteins is not obvious. Noroviruses contain evolutionarily conserved RNA structures, which when disrupted, reduce or completely destroy replication of MNV [127,128]. Evidence suggests HuNV ProPol can catalyze nucleotidylylation independently of a poly (A) RNA template in the presence of Mn^2+^ as a divalent metal cation, whereas mature polymerase is more active in the presence of an RNA template, regardless of the cation available [4,27,129]. Uridylylation by the HuNV ProPol, which does not strictly require an RNA template, can nevertheless be enhanced upon addition of an ORF3-3′ UTR poly (A) template [27]. A similar stimulatory effect on nucleotidylylation activity has also been shown for the MNV polymerase [130]. FCV ProPol, unlike HuNV ProPol, is strictly dependent on the presence of an RNA template as a cofactor for nucleotidyl-transfer [26,123].

#### 4.1.3. Nucleotide Selection for Nucleotidylylation

The HuNV MD145 strain VPg has been shown to be preferentially nucleotidylylated with GTP and UTP over other nucleotides, exhibiting a 2-fold preference for GTP over UTP [27]. A similar finding was found by Medvedev et al. [121], and has also been shown for MNV VPg, which is preferentially guanylylated compared to incorporation of the other NTPs [130]. As all calicivirus genomic and subgenomic RNAs begin with a guanine nucleotide, this supports the priming theory of positive-sense viral RNA replication by a guanylylated VPg.

Both UTP and GTP may be added to VPg, and some evidence suggests that two nucleotides can be added to calicivirus VPg proteins [27], similar to the VPgpUpU formed during picornavirus replication. The VPg of RHDV was originally proposed to be uridylylated by the RNA polymerase [7], based on analogies to picornavirus VPg. Belliot et al. [27] proposed that uridylylated VPg may prime initiation of RNA synthesis only on the anti-sense genome or anti-sense subgenome on the poly (A) tail. Using recombinant polymerase and subgenomic RNA as a surrogate for the norovirus genome, Rohayem et al. showed that uridylylated HuNV VPg primed the subgenome in vitro [4], but not the anti-subgenome. This was consistent with experimental observations in FCV and MNV, which did not find that the antigenome was VPg-linked [26,131], and with findings that initiation of replication of the antigenome occurs via a primer-independent *de novo* mechanism [4]. If VPg does prime antisense genomic RNA synthesis, then a role for uridylylated VPg can be proposed for this process. That GTP is preferred over UTP would be expected in this case, given that the antisense genome is >1000-fold less abundant than the positive-sense genome during viral replication [131].

To date, the mechanisms behind nucleotide discrimination in nucleotidylylation remain unclear. An NTP-binding sequence has been identified in calicivirus VPgs [121], suggesting VPg itself, as opposed to the polymerase, may bind NTPs prior to nucleotidylylation, and the nucleotide addition cycle of nucleotidylylation begins with the binding of VPg-NTP by the polymerase. However, despite the fact that VPg is preferentially guanylylated, VPg was shown to bind all NTPs with equal efficiency [121], suggesting that the polymerase may have a discriminatory role in preferential NTP addition. It may be possible that VPg is both guanylylated and then uridylylated, as the second nucleotide of norovirus genomes is a uracil. However, how a specific VPgpGpU might be synthesized is unclear, although it is interesting to speculate on the role an RNA template might have in this process, and whether the nucleotides might be added by different forms of polymerase.

## 5. Potyviruses

Potyviruses are a genus of plant viruses belonging to the *Potyviridae* viral family and have a wide range of plant hosts, including many vegetables and legume species which constitute important parts of the global food supply. Potyviruses are some of the most common plant viruses in the world, accounting for approximately ~30% of known plant pathogens. Potyviruses are known for causing extensive economic damage to important crops such as potatoes, turnips, tomatoes, beans and peas. Potyviruses are primarily transmitted from plant to plant by arthropods [132]. The viral particles are filamentous and almost all members of the *Potyviridae* have a monopartite +ssRNA genome of 8–11 kb in length. Potyvirus genomes encode an ORF which is translated to a 340–370 kDa polyprotein, which is then co- and/or post-translationally processed by three forms of internally encoded protease into cleavage intermediates or mature protein products (Figure 1). A fusion protein, P3N-PIPO, is read off an alternative open reading frame as a result of a frameshift caused by RNA polymerase slippage on the first ORF [133,134].

### Potyvirus Nucleotidylylation

Potyvirus VPg proteins range from 20–22 kDa and exist in both a mature form as well as a number of uncleaved precursor forms. The potyvirus turnip mosaic virus appears to possess VPg exclusively in precursor forms, as one study focusing on subcellular localization could not detect the mature form of VPg [135]. Potyvirus VPgs, like other viral VPgs, have an ordered central domain flanked by disordered N- and C-termini [136,137]. VPgs from *Potyviridae* share roughly 50% sequence identity, mostly in the conserved regions of the NTP binding motif, and a bipartite nuclear localization signal [138].

Potyvirus VPgs are genome-linked to the 5′ end of the viral nucleic acid and prime the synthesis of the viral genome [139,140]. As with caliciviruses, potyvirus VPg proteins are covalently bound to genomic RNA by a conserved tyrosine residue. In tobacco vein mottling virus (TVMV) VPg is linked via Tyr60 to the viral nucleic acid, whilst pepper vein banding virus (PVBV) VPg is linked via Tyr66 and potato virus A (PVA) VPg is linked by Tyr63 and possibly by an alternative residue Tyr119 [6,141,142]. In addition to functioning as a genome cap, potyvirus VPgs share a number of other functional similarities to VPgs from picornaviruses and caliciviruses, including binding to host factors such as eIF4E [143]. Potyvirus VPgs are also uridylylated by their respective RNA polymerases [6,138,141].

Co-purification of TVMV VPg with a GST-labelled polymerase fusion protein demonstrated that VPg associates with the polymerase in vitro [144]. Tyr60 of the VPg was shown to be essential for this interaction, as mutation of this residue ablated the association between the polymerase and the VPg entirely [145], preventing viral replication in protoplasts [28,142]. Further, TVMV VPg stimulates inherent polymerase activity, acting as a co-factor in RNA synthesis in addition to the role of VPg as a primer of the viral RNA genome [144].

Studies of PVA and PVBV VPg uridylylation showed that RNA polymerase uridylylates VPg in the presence of a divalent metal cation independently of a poly (A) RNA template [6,141]. A conserved tyrosine residue in PVBV VPg, specifically Tyr42, is part of the nucleotide binding motif AYTTKKGK [141], analogous to a nucleotide binding motif in closely related PVA VPg [6]. However, deletion of this nucleotide binding motif in PVA VPg did not result in complete loss of uridylylation [6], indicating that Tyr42 was not the nucleotidylylation target residue of the PVA polymerase. Tyr66 was identified as the target residue when mutation of Tyr66 to threonine prevented uridylylation by PVBV RNA polymerase [141]. It is not clear if potyvirus VPgs are nucleotidylylated with one residue or two, whether there is nucleotide preference, and whether potyvirus precursor polymerases outperform mature polymerases in this function.

## 6. Coronaviruses and Other Nidoviruses

The order *Nidovirales* encompasses 14 families of positive-sense RNA viruses, within which 109 species have currently been identified [146]. Of this order, three families of biological and economic value are *Arteriviridae*, *Mesoniviridae* and *Coronaviridae*. Arteriviruses are known to infect a diverse group of mammals including pigs (porcine reproductive and respiratory syndrome virus; PRRSV), horses (equine arteritis virus; EAV) and monkeys (simian hemorrhagic fever virus) [147]. PRRSV is estimated to cause $550 million in losses in the US alone, making it a pathogen significant to the agricultural industry [148]. Viruses within the *Mesoniviridae* family infect mosquitos, and due to the importance of mosquitos in vector transmission, it is an area that warrants further research [149]. Coronaviruses are the causative agents for three major disease outbreaks: SARS (severe acute respiratory syndrome) caused by the SARS virus, MERS (Middle East respiratory syndrome) caused by the MERS virus and COVID-19 (coronavirus disease 2019) caused by SARS-CoV-2, all of which have arisen from animal reservoirs [150]. General pathologies include pneumonia, fever and dry cough, with other symptoms being more strain specific. Four other species of coronavirus have been found in humans (HCoV229E, HCoV-OC43, HCoV-NL63 and HCoV-HKU1) and are estimated to be responsible for 15–30% of all cases of the common cold worldwide [151]. Other viruses of interest in the *Coronaviridae* family include toroviruses, which primarily infect livestock, although some strains have been identified to cause disease in humans [152,153].

### 6.1. Nidovirus RdRp and Nucleotidylylation

The RdRps of coronaviruses (nsp9 for EAV or nsp12 for HCoV-229E, SARS or SARS-CoV-2) are made up of three main domains: the RdRp domain, the nidovirus RdRp-associated nucleotidyltransferase (NiRAN) domain and an uncharacterized third domain that connects the NiRAN to the RdRp domain [36]. In vitro studies using the SARS nsp12 show that it can perform primer-dependent and primer-independent RNA synthesis [154]. Separately, nsp12 has poor polymerase activity, but when in a complex with nsp7 and nsp8, the processivity of RNA synthesis is increased [155].

While coronaviruses do not possess a VPg protein as described for other viruses in this review, they perform nucleotidylylation as a critical component of the viral replication cycle. Currently, there is only experimental data on nidovirus nucleotidylylation from four viruses: EAV, HCoV-229E, SARS and SARS-CoV-2. As with the other ‘branch 2′/picornavirus supergroup of viruses [14] described earlier in this review, nucleotidylylation involves the polymerase linking a nucleotide to a separate viral protein or to itself.

The preference of the incorporated nucleotide during nucleotidylylation by SARS-CoV-2 RdRp and HCoV-229E RdRp is UTP over GTP with the HCoV-229E activity being 2–3-fold higher using UTP [156]. However, for EAV, this preference for UTP appears to be pH-dependent, with the nucleotide preference switching to GTP above pH 8.5. Furthermore, characterization of the EAV RdRp activity identified that GTP was able to outcompete UTP for nucleotide preference in a competition assay [37]. The modified residue was shown to be K380 via mass spectrometry, and this was validated by mutation of the site resulting in no nucleotidylylation.

#### The NiRAN Motif

**Figure 3:**
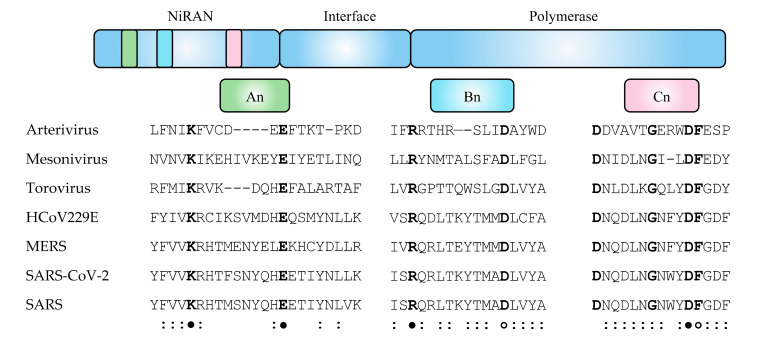
Sequence alignment of the NiRAN domains from viruses within the order *Nidovirales*. Schematic diagram of nsp12 (nsp9 for Arterivirus). Sequence alignment of the N-terminal region of the RdRps from EAV (Arterivirus NP_705590.1), Dak Nong virus (Mesonivirus YP_009505590.1), Breda virus (Torovirus YP_337905.2), HCoV229E (QNT54752.1), MERS virus (YP_009047223.1), SARS-CoV-2 (QHD43415.1) and SARS virus (AER30332.1) was generated by Clustal Omega [158]. The three NiRAN motifs are designated A_n_, B_n_ and C_n_. Bolded resides denote invariant residues and a colon (:) indicates a residue that is conserved in more than 50% of the viruses aligned. The filled circles indicate positions that are lethal when mutated, and open circles indicate residues that result in impaired virus when mutated.

Analysis of nidovirus polymerase sequences has identified a unique domain in the N-terminal portion of nidovirus RdRp, termed a NiRAN domain. Alignments of this domain between viruses from *Coronaviridae*, *Toroviridae*, *Mesonivirdae* and *Arterivirdae* families [36] showed several areas of conservation indicating that the NiRAN domain is found in these different viral lineages. Closer analysis of this N-terminal domain of the RNA polymerase identified eight invariant residues, which were further mapped to three motifs denoted A_n_, B_n_ and C_n_ (Figure 3). Mutational analysis of these invariant residues has shown either severe growth impairment or a nonviable virus [36,156]. The NiRAN domain is considered to be a hallmark of *Nidovirales*, with no homologs identified elsewhere [36,157]. Whether the other viral RdRps that perform protein nucleotidylylation have alternate conserved motifs critical for this function is unknown.

### 6.2. Nucleotidylylation of Nidovirus Proteins

#### 6.2.1. EAV nsp7

Each of the RdRps described above were identified to nucleotidylylate other non-structural proteins. The nucleotidylylation activity of EAV RdRp was notably enhanced when in the presence of nsp7 [37]. Nsp7 is unique to the *Arteriviridae* family [159] and is processed by the EAV protease into two proteins, nsp7α and nsp7β [160]. The role in the viral life cycle has not been elucidated for EAV; however, studies in PRRSV show that nsp7 is essential for virus survival and indicate that it may have a role in RNA synthesis [161]. Three sites in nsp7β (K143, K156 and K172) were shown to be guanylylated by the RdRp. Two of these sites (K156 and K172) were seen to be conserved in PRSSV [37]. The role these modifications have in EAV is unknown.

#### 6.2.2. Coronavirus nsp9

The nsp9 of both HCoV-229E and SARS-CoV-2 are nucleotidylylated by their respective RdRps. Both viruses preferentially use UTP as a substrate; however, GTP can be utilized [37,156]. Nsp9 is a small dimerized protein [162] that has been shown to be critical for growth in SARS [163]. Sequence analysis has shown nsp9 is likely to be unique to the coronavirus family, with large regions of conservation between coronaviruses, and is able to bind to ssRNA and ssDNA [164]. Characterization of the interaction between RdRp and nsp9 showed that for nucleotidylylation to occur, the N-terminal end of nsp9 had to be free from the pp1ab polyprotein, and it is proposed that the interaction between the nsp12 and nsp9 is transient [156]. Tandem mass spectrometry identified Asn1 of the HCoV-229E nsp9 to be modified, and it is proposed that the linkage occurs on the primary amine of the amino acid. Of particular note, mutational analysis showed that the nucleotidylylation activity was independent of the RdRp activity [156].

#### 6.2.3. Coronavirus nsp7 and nsp8

While HCoV-229E RdRp was not seen to nucleotidylylate nsp7 in an in vitro assay [156], the SARS-CoV-2 RdRp was able to link a GTP to K2 of the nsp7 via a phosphoramide bond [37]. Further mutation of K2 significantly reduced the amount of radiolabeling seen on nsp7, indicating that this is likely to be the preferred nucleotidylylation site [37]. Both GTP and UTP are linked to nsp7, with GTP being preferred over UTP during nucleotidylylation; however, UTP was able to label nsp7 and nsp8 more effectively, possibly due to UMP being more stable than GMP. Nsp8 was also seen to be labelled with UTP and GTP; however, the site of modification is not known [37]. Nsp7 and nsp8 of SARS-CoV-2 are able to form a heterotetramer structure proposed to have primase activity [165], which has been seen to act as a co-factor for the RdRp, increasing its polymerase activity [166]. The role of nucleotidylylation in SARS-CoV-2 is unknown.

### 6.3. Role of Nucleotidylylation

Nucleotidylylation activity is essential for coronavirus replication, with nucleotidylylation knockout mutations resulting in a nonviable virus. Slanina et al. [156] proposed that the nucleotidylylation of the coronavirus nsp9 would allow it to act as a protein primer for RNA synthesis. This is supported by the RdRp from both the SARS virus and EAV being able to perform de novo and protein-primed RNA synthesis [2,36]. The RNA binding activity of nsp9 lends credibility to this proposal; however, since no nsp9 linked to RNA has been identified, there would have to be an additional mechanism that releases the nsp9 from the RNA. It has also been suggested that UMP linked to the coronavirus nsp9 interact with cis-acting RNA elements identified at the 3′ end on the coronavirus genome [156].

Yan et al. showed that the NiRAN domain of nsp12 is able to catalyze the transfer of GMP to ppA, which forms the GpppA cap core structure. They suggested that the association of the nsp9 to the NiRAN domain may stabilize the 5′ end of GpppA-RNA due to nsp9 being an RNA-binding protein before the GpppA-RNA is further processed during cap synthesis [167].

## 7. Conclusions

Protein nucleotidylylation is a critical component of the replication of a range of viruses, with this modification commonly found in +ssRNA viruses of the picornavirus supergroup [14], and highlights a common feature of this group of viruses. These viruses are important pathogens of humans, other animals and plants with a significant impact on health and well-being, as evidenced by the current SARS-CoV-2 global pandemic. Nucleotidylylation is central to the initiation of viral genome replication, an essential step in the viral life cycle, and the placement of caliciviral and potyviral VPg proteins at the 5′ end of the viral genome is critical for the recruitment of translational initiation factors, and hence, viral protein synthesis, for these two viral families. While nucleotidylylation plays a central role in the replication of viruses that perform this function, the underlying mechanisms and processes largely remain to be determined.
